# Investigating the Gut Microbiota Composition of Individuals with Attention-Deficit/Hyperactivity Disorder and Association with Symptoms

**DOI:** 10.3390/microorganisms8030406

**Published:** 2020-03-13

**Authors:** Joanna Szopinska-Tokov, Sarita Dam, Jilly Naaijen, Prokopis Konstanti, Nanda Rommelse, Clara Belzer, Jan Buitelaar, Barbara Franke, Mirjam Bloemendaal, Esther Aarts, Alejandro Arias Vasquez

**Affiliations:** 1 Department of Psychiatry, Radboudumc, Donders Institute for Brain, Cognition and Behaviour, 6525 GA Nijmegen, The Netherlands; Joanna.Szopinska-Tokov@radboudumc.nl (J.S.-T.); Nanda.Lambregts-Rommelse@radboudumc.nl (N.R.); Barbara.Franke@radboudumc.nl (B.F.); Mirjam.Bloemendaal@radboudumc.nl (M.B.); 2 Department of Cognitive Neuroscience, Radboudumc, Donders Institute for Brain, Cognition and Behaviour, 6525 EN Nijmegen, The Netherlands; Sarita.Dam@radboudumc.nl (S.D.); jilly.naaijen@donders.ru.nl (J.N.); Jan.Buitelaar@radboudumc.nl (J.B.); 3 Laboratory of Microbiology, Wageningen University, 6708 WE Wageningen, The Netherlands; prokopis.konstanti@wur.nl (P.K.); clara.belzer@wur.nl (C.B.); 4 Karakter Child and Adolescent Psychiatry University Center, 6525 GC Nijmegen, The Netherlands; 5 Department of Human Genetics, Radboudumc, Donders Institute for Brain, Cognition and Behaviour, 6525 GA Nijmegen, The Netherlands; 6 Centre for Cognitive Neuroimaging, Donders Institute for Brain, Cognition and Behaviour, Radboud University, 6525 EN Nijmegen, The Netherlands; esther.aarts@donders.ru.nl

**Keywords:** gut microbiota, ADHD, 16S rRNA gene, inattention

## Abstract

Attention-deficit/hyperactivity disorder (ADHD) is a common neurodevelopmental disorder. Given the growing evidence of gut microbiota being involved in psychiatric (including neurodevelopmental) disorders, we aimed to identify differences in gut microbiota composition between participants with ADHD and controls and to investigate the role of the microbiota in inattention and hyperactivity/impulsivity. Fecal samples were collected from 107 participants (N_ADHD_ = 42; N_controls_ = 50; N_subthreholdADHD_ = 15; range age: 13–29 years). The relative quantification of bacterial taxa was done using 16S ribosomal RNA gene amplicon sequencing. Alpha and beta-diversity were not different between participants with ADHD and healthy controls. Three genera showed nominal differences (*p*_uncorrected_ < 0.05) between both groups (*Prevotella_9*, *Coprococcus_2* and *Intestinibacter*) and were further tested for their association with ADHD symptom scores (adjusting for age, sex, body mass index, a time delay between feces collection and symptoms assessment, medication use and family relatedness). Our results show that the variation of a genus from the *Lachnospiraceae* family (*Coprococcus_2*) showed a trend of being negatively associated with inattention symptoms. Furthermore, we showed that the relative abundance of four genera was reduced by ADHD medication (*p*_uncorrected_ < 0.05). Overall, our results may support the role of the gut microbiota in the pathophysiology of ADHD. Given the scarcity of studies on the gut microbiota in individuals with ADHD, the current results are an important contribution to this field. More studies are needed into the gut microbiota as part of the pathology of ADHD, especially with a bigger sample size across the lifespan and more detailed information about lifestyle.

## 1. Introduction

Attention-deficit/hyperactivity disorder (ADHD) is a common, highly heritable [1], heterogeneous neurodevelopmental disorder with around 5% prevalence in children and 2.5% prevalence in adults worldwide [2]. The disorder is characterized by age-inappropriate levels of inattention and/or hyperactivity and impulsivity. ADHD has a significant social impact on patients’ lives, causing disruption at school [3], work [4], and in personal relationships [5]. Typically, ADHD has its onset in childhood and can persist into adulthood. ADHD is considered a multifactorial disorder with multiple (common and rare) genetic variants, in combination with the environment, explaining its etiology and phenotypic variation [6].

Treatment of ADHD usually involves prescription of either stimulant or non-stimulant medication that target specific systems related to dopamine, noradrenaline, and/or serotonin neurotransmission [7]. These pharmacological interventions are highly effective in controlling ADHD symptoms and have an approximate response rate of 70% [8]. However, medication treatment of ADHD is limited by low adherence, concerns about side effects, and absence of evidence for long-term efficacy [9,10]. Approximately 30% of individuals with ADHD do not respond to medication or are unable to tolerate the adverse effects [7]. For (some of) those patients, non-pharmacological treatments are a suitable alternative [11].

One of the non-pharmacological interventions recently suggested to influence ADHD symptom severity is diet (e.g. elimination diet) [12,13]. Diet can exert its effects on ADHD through the gut-brain axis. The gut-brain axis is a continuous and bidirectional communication system between the enteric and central nervous systems including the cognitive and emotional centers of the brain [14]. The gut-brain axis has been suggested to modulate the risk for several psychiatric illnesses, including ADHD [15,16], and can influence cognitive processes, mood, and brain performance [17].

A key player in the gut-brain axis is the complex ecosystem of commensal bacteria living in our gut, the microbiota [18]. DNA sequencing makes it possible to investigate the microbial composition and its potential role(s) in the risk and pathophysiology of ADHD [19]. For example, in our previously published study, we observed significantly enhanced predicted microbial biosynthesis of phenylalanine (a dopamine precursor related to an increase in the genus *Bifidobacterium*) associated with a neural hallmark of ADHD, for instance, decreased functional responses of the ventral striatum during reward anticipation [19].

In the present study, we increased the sample size of Aarts et al. (16) (samples overlap ~40%) in order to investigate (i) if there are differences in gut microbiota composition between participants with ADHD and age matched controls, and if so, (ii) whether these bacteria are associated with the severity of ADHD symptoms.

## 2. Material and Methods

### 2.1. Study Participants

To evaluate gut microbiota composition in participants with ADHD and healthy controls, we collected fecal samples from 107 Dutch (Caucasian) individuals enrolled in the follow-up of the NeuroIMAGE study [20]. Three groups were included: participants with ADHD (*N* = 42), subthreshold ADHD (*N* = 15; participants who did not reach the criteria for being considered as ADHD but scored too high to be considered healthy control; since we divided the analysis between case/control and continuous analysis, the subthreshold ADHD group was excluded from case–control comparisons), and healthy controls (*N* = 50). The cohort included sibling pairs, which was taken into account in the analysis. A semi-structured diagnostic interview of DSM-IV criteria was conducted with both the participant and his/her parents using the Kiddie-Schedule for Affective Disorders and Schizophrenia (K-SADS) according to DSM-IV criteria. Clinical diagnosis was confirmed using a diagnostic algorithm which combined the diagnostic interview (K-SADS) with the Conners rating scales [20].

Continuous measures of inattention severity (IA) and hyperactivity/impulsivity severity (HI) were derived from the Conners Adult ADHD Rating Scales (CAARS; ≥ 16 years) and Conners Teacher Rating Scale (CTRS; < 16 years). For participants using medication, ratings were based on each participant’s functioning off medication. Detailed recruitment and diagnostic information can be found in the NeuroIMAGE design article [20].

Additionally, the following information was obtained from medical records: age, sex, Body Mass Index (BMI), time delay between feces collection and symptom assessment (differences in days, further on called “diff_days”), and use of ADHD-related medication. Five samples with missing BMI values were excluded from the regression analysis. For an overview of the participant characteristics, see Table 1. Information regarding the use of ADHD medication was provided via self-report (≥16 years) or parental report (<16 years) on the day of measurement. Two controls were removed because they indicated the use of ADHD medication.

Ethical approval for the study was obtained from the local research ethics committees (Commissie Mensgebonden Onderzoek (CMO) Regio Arnhem-Nijmegen) on 9 April 2013 and can be found under registration number 2012/542 and ethics protocol number NL nr.: 41950.091.12. A written informed consent was obtained from all participants and/or their parents prior to the sample and data collections.

### 2.2. Microbiota Methods and Measures

#### 2.2.1. Sample Collection, Preparation and Sequencing

The human fecal samples were collected at home by the participants and stored at 4 °C. Within 24 hours after collection, the samples were transported to the laboratory, aliquoted into 1.5 ml Eppendorf tubes, and stored at −80 °C. The bacterial DNA was extracted using a repeated bead-beating step and the Maxwell® 16 Instrument (Promega, Leiden, The Netherlands), as described previously [21]. DNA purification was performed with a customized kit (AS1220; Promega, Leiden, The Netherlands). The purified bacterial DNA was measured with a NanoDrop ND-2000 spectrophotometer (Thermo Fisher Scientific, Wilmington, DE, USA), and aliquots of 20 ng/µL were prepared for the 2-step Polymerase Chain Reaction (PCR) reactions (including negative controls). In the first PCR, amplification of the V1-V2 region of the 16S rRNA gene was performed using previously reported primers for this region: 27F-DegS (5’GTTYGATYMTGGCTCAG) – 338RI-II (5’ GCWGCC[T/A]CCCGTAGG[A/T]GT) [22]. In the second PCR, unique barcoded primers were added to each sample to allow for parallel sequencing of many different samples. The PCR product was checked using electrophoresis and purified using the CleanPCR kit (CleanNA, Alphen aan den Rijn, The Netherlands). The DNA concentration was measured using Qubit® 2.0 fluorometer. The purified samples were used to prepare libraries for the Illumina HiSeq PE300 sequencing platform (GATC Biotech AG, Konstanz, Germany), with final loading concentrations of 200 ng/µL.

#### 2.2.2. Data Processing

The sequenced data was analyzed through NG-Tax 16S rRNA pipeline at Wageningen University and Research (WUR, Wageningen, The Netherlands) [23]. NG-Tax identified the taxonomy of the samples based on 16S sequences using three core elements: (i) barcode-primer filtering, (ii) operational taxonomic unit (OTU) picking, in which unique sequences with the relative abundance above 0.1% were clustered into OTUs based on a sequence similarity ≥98.5%, and (iii) taxonomic assignment using the SILVA reference database (version 128; [24]).

#### 2.2.3. Filtering Procedure of Taxonomic Data

We performed two filtering steps on the output file (BIOM-file) of NG-Tax in order to remove genera with low prevalence and to reduce the impact of the high number of absent genera (with a value of zero per sample). This step was critical to improve the power to detect the true effects of the microbiota while keeping as much information as possible. The way the genera/OTUs were identified made it impossible to disentangle if the observed values of zero correspond to true zeros (e.g. not present in the sample) or are false zeros (e.g. present but not detected). The two filtering steps were applied as follows: (i) the OTU table was filtered at the genus level, where a genus with non-zero values in less than 10% of the samples was removed, and (ii) the OTU table was filtered at sample level, at which a sample with less than 10% of genera was removed (Figure S1). The results of 16S rRNA analysis after filtering of taxonomic data can be found in the Supplemental Results.

#### 2.2.4. Sequencing Depth Comparison

The sequencing depth of the microbiota data was compared between all groups (ADHD, controls and subthreshold ADHD) by performing a Kruskal–Wallis H test on the total reads. This was done in order to assess equal distribution of the sequence reads across the groups, which helped to verify the effect of any technical variation between the groups. The results can be found in the Supplemental Results, which indicated no differences.

#### 2.2.5. Within-Sample Diversity Metrics

Three alpha-diversity metrics were applied on the OTU level: (1) the species richness estimator, counting the observed unique OTUs in each sample [25]; (2) Shannon–Wiener diversity [25] index, which takes into consideration not only the number of observed unique OTUs but also their abundance; and (3) the phylogenetic richness estimator, which estimates microbial diversity across a phylogenetic tree (Faiths’ phylogenetic diversity) [26]. The alpha-diversity metrics were calculated using the R function microbiome::alpha (version 1.6.0) [27] and compared between participants with ADHD and controls

#### 2.2.6. Between-Sample Diversity Metrics

To assesses beta-diversity, we used the weighted UniFrac distance metric, a phylogenetic-based assessment of the difference in overall bacterial community composition at the OTU level [28]. To analyze the beta-diversity, multivariate statistics were conducted using ADONIS and betadisper functions in the R package vegan version 2.5–2 [29,30]. Through ADONIS, we determined if the tested variables (i.e. disease status or symptom counts) influenced beta-diversity [29]. Betadisper measures the variability in OTU composition among groups (here ADHD and controls) [31]. Principal Coordinates Analysis (PCoA) was performed using the R function phyloseq::ordinate to determine and visualize whether there is a clear discrimination of microbial composition between the two groups.

#### 2.2.7. Taxonomic Composition Analysis and Associations with Symptoms

Taxonomic composition of the gut microbiota was investigated at the phylum and genus levels after transforming the sequencing read counts into microbial relative abundance (normalization step). Any unknown taxonomic level (e.g. unknown genus) was assigned to the next highest known taxonomic rank (e.g., family). The composition analysis was calculated using phyloseq R package version 1.28.0 [32]. Microbiota compositional data are highly skewed given the high number of zeros. We used non-parametric statistics (which is less sensitive to the extreme values), in our case the Mann-Whitney U test, to identify (nominal) statistical differences in the relative abundance of gut microbiota between participants with ADHD and controls. This was visualized by using a boxplot with a summary table representing the number of zeros using “ggpubr” R package version 0.4.0.999. To prioritize the selection of candidate taxa without making any claims of association (with ADHD), genera showing nominal statistical differences (*p* < 0.05, uncorrected) were selected for downstream correlation and linear regression analyses.

Linear mixed regression analysis was performed to associate bacterial relative abundance with inattention or hyperactivity/impulsivity score available for all participants (including participants with “subthreshold ADHD”). Models were adjusted for age, sex, BMI, diff_days, and included the family relatedness as random factor; this was done by using the R function lme4::lmer. Given the skewed distribution of the microbial relative abundance prior to the association analyses, we investigated linear regression assumptions and identified and removed extreme and influential samples (outliers). Outliers are known to have a significant effect on the regression model (but not on the non-parametric test [33]). Outliers were defined by Cook’s distance above (4/n) (where n is the number of observations) and Leverage value above (3 × (k + 1)/n) (where k is the number of independent variables, in our case k = 5) [34,35]. Cook’s distance identifies influential values, which do not have to be necessarily the extreme ones; these can be identified by Leverage. Therefore, a sample was excluded from the analysis only if it scored above the threshold for both values. Regression analyses were corrected for multiple testing using the false discovery rate (FDR; in total corrected for 6 tests) and indicated as q-values (Q).

#### 2.2.8. Effect of Medication on the Regression Results and on Gut Microbiota Composition

Often ADHD patients are medicated, and some studies show that medication can influence gut microbiota composition [36,37]. Therefore, we explored an effect of ADHD medication on our (regression) results and on gut microbiota composition at the genus level. The regression model could not simply be adjusted for medication due to the large number of non-medicated cases, who do not equate to healthy controls. Thus, the medicated cases (*N* = 19) were removed from the regression model to see how this effects the results.

#### 2.2.9. Correlation Analysis and Multiple Regression with All Selected Genera

The gut microbiota is a highly complex ecosystem of interacting organisms. In order to investigate the (in)dependent effect of the selected genera on symptoms, we investigated their correlation structure and performed multiple regression analysis. The genus-genus correlation was assessed based on Spearman’s rank correlation coefficient. Multiple regression analysis was performed for the same selected genera tested in the univariate models, adjusting for age, sex, BMI, diff_days, and family relatedness as a random factor; the analysis was done without the samples identified as outliers (see above). The results are shown in supplementary materials. 

Across all the paper, the (non)parametric analyses were preceded by the Shapiro-Wilk’s normality test using stats package version 3.6.3. Visualizations were created by using ggplot2 (version 3.3.0) and ggpubr (version 0.4.0.999) packages. 

## 3. Results

### 3.1. Subjects Characteristics

The general characteristics of the studied sample are presented in Table 1. Mean age, median BMI, percentage of males, and differences in days between fecal collection and ADHD symptoms assessment (diff_days) were similar among the two groups. As expected, mean inattention and hyperactivity/impulsivity scores were statistically different between the ADHD and control groups. Out of the 41 participants with ADHD, 19 were using medication for ADHD. 

### 3.2. Microbiota Measures

*Within- and between-sample diversity metrics:* None of the three alpha-diversity (within-sample diversity) measures showed significant differences between the ADHD and control groups (Figure S2). 

**Figure 1:**
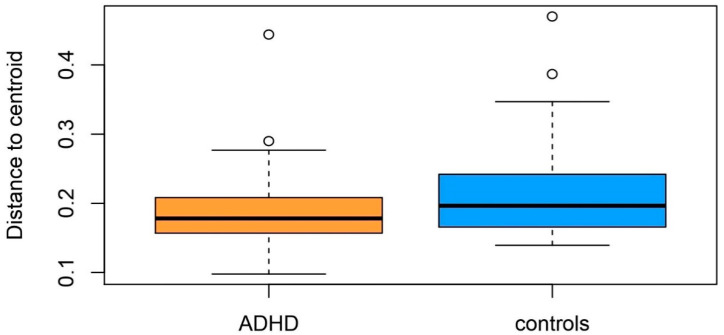
Boxplot of multivariate homogeneity of groups’ dispersions (betadisper) of participants with ADHD and controls. Box plots represent median with whiskers on ±1.5 IQR. Pseudo-F = 3.051, *p* = 0.08.

Beta-diversity (between-sample diversity), assessed using betadisper [30], showed that the ADHD group had a smaller variation in the gut microbiota composition (*p* = 0.08; Figure 1 and Figure S3). PCoA based on weighted UniFrac distance did not show discrimination of microbial composition between the two groups determined by disorder status (ADHD vs. controls) (Figure S3). This was supported by the statistical test—ADONIS, where participants with ADHD and controls samples displayed non-significant separation according to weighted UniFrac distance (variance explained = 0.9%, *p* = 0.479, *N* = 89). Other variables, such as age, sex, BMI, inattention score (IA), hyperactivity-impulsivity score (HI), and medication, did not show a significant effect on beta-diversity (Table 2). 

#### 3.2.1. Taxonomic Composition Analysis and Associations with Symptoms

As expected from [38], a compositional analysis of our samples revealed that *Firmicutes*, *Bacteroidetes*, *Actinobacteria*, *Proteobacteria*, and *Verrucomicrobia*, were the most frequent phyla in our data (Table S2). There were no significant differences in the relative abundance of any of these phyla between participants with ADHD and controls (Table S2).

**Figure 2:**
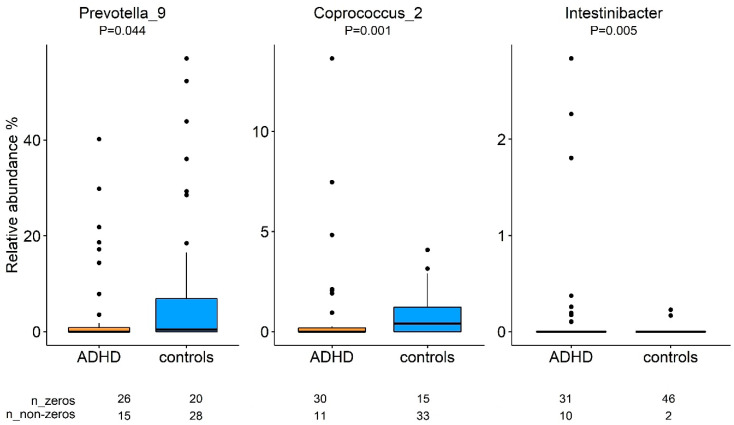
Comparison of bacterial relative abundance between participants with ADHD and controls. Identification of the bacteria differences was made by the Mann-Whitney test. No outliers were removed since we used a non-parametric test which is less sensitive to the extreme values. Box plots represent median with whiskers on ±1.5 IQR. Nominal significant threshold: *p* < 0.05.

At the genus level, differences in the gut microbiota composition revealed nominal significant case-control differences for three genera (*p* < 0.05; Figure 2). Of those, one genus was higher, and two were lower in participants with ADHD compared with control samples. One genus, *Coprococcus_2* showed a trend of being negatively associated (B = (−3.189), *p* = 0.055, Q = 0.33; corrected for multiple testing; Table 3) with inattention scores. We did not find any association between tested genera and hyperactivity/impulsivity scores (before or after correcting for multiple testing; all *p* > 0.05); therefore, only IA was considered in further analyses.

#### 3.2.2. Effect of Medication on the Regression Results and on Gut Microbiota Composition

We tested the effect of ADHD medication on the (regression) results by excluding medicated cases (*N* = 19) from the analysis. We found that medication reduced the beta coefficient from −3.189 to −2.806 in the association between *Coprococcus_2* and symptoms of inattention (B = (−2.806), *p* = 0.080 vs. results in Table 3). This reduction can be due to the reduction in sample size (*N* = 79 vs. *N* = 95).

We performed a post hoc exploratory analysis where we compared the relative abundance of all the genera (total taxa compared = 77) between the medicated (*N* = 19) vs. non-medicated (*N* = 22) individuals with ADHD. We found that four genera (*Lactobacillus*, *Lachnospiraceae_ND3007_group*, *Ruminococcaceae_g__* and *Ruminococcaceae_UCG.014*) were decreased in medicated ADHD (*p*_uncorrected_≤ 0.05; Figure S4). Regarding the *Lactobacillus* results, we had to treat them with caution because we only had three non-zero values for medicated cases.

## 4. Discussion

This study aimed to determine the differences in gut microbiota composition between individuals with ADHD and controls and the association between the abundance of the selected genera and the severity of ADHD symptoms (inattention and hyperactivity/impulsivity) accounting for the effects of medication. Our results did not show general differences in microbiota composition (beta-diversity) between the groups. At the taxonomic level, we found nominal (uncorrected significant) differences at the genus level; lower abundance of *Prevotella_9* and *Coprococcus_2* and higher abundance of *Intestinibacter* in individuals with ADHD compared to controls. Of these three genera, *Coproccocus_2* related most strongly (*p* = 0.055) with ADHD symptoms, specifically Inattention symptoms. Excluding subjects that were using ADHD medication from the regression model slightly reduced the strength of the association. Together this indicates that differences in gut microbiome in this sample of ADHD patients compared with control subjects are subtle.

Our results align with the growing evidence that gut microbiome alterations might be part of the pathology of ADHD [19,39,40,41,42]. The taxa, observed to be nominally different, partly overlap with previous findings. For example, while not the genus showing the largest differences, Aarts et al. also found the genus *Coprococcus* to be underrepresented in individuals with ADHD [19]. Our lab recently performed a humanization study, in which six randomly selected microbiome samples from the NeuroIMAGE cohort (the cohort studied here) were transplanted into germ-free wild-type mice [43]. Mice colonized with ADHD gut microbiota had increased anxiety-like behavior and showed significantly altered structural and functional brain characteristics. When comparing taxonomy between cases and controls in this humanization approach, again, *Coprococcus_2* was found altered. Here, the effect was in the opposing direction; relative abundance was increased in mice colonized with ADHD gut microbiota, wherein the current case-control comparison *Coprococcus_2* abundance was higher in controls. Putting aside differences in the directions of effects, the fact that genus *Coporococcus_2* surfaces in both case-control comparisons suggest that this is an interesting target for replication in gut microbiota associated with ADHD diagnosis.

Furthermore, an abundance of the genus *Prevotella* was also found lower in children with ADHD compared with controls [39]. Functionally, *Prevotella* spp. and some *Coprococcus* species have been identified as short-chain fatty acids (SCFAs) producers [44], which can be absorbed and used as an energy source by the host [45]. SCFAs producers have been shown to play a potential role in ADHD [46] and autism [47,48] through several of the gut-brain-routes, including their anti-inflammatory effects on the central nervous system.

The only genus with a higher rather than lower relative abundance in cases versus controls, *Intestinibacter* (belonging to *Peptostreptococcaceae*), was defined only recently [49]; not much is known about its role in ADHD and human health in general. A potential function may be involved in mucus degradation [50]. Mucus-degrading bacteria are linked to inflammatory bowel disease [51], a comorbid diagnosis seen in neurodevelopmental disorders like ASD [52] or ADHD [53]. Note that the relative abundance of this genus is quite low in both groups, and the statistical difference is based on ten non-zero observations in the ADHD group versus two non-zero observations in the control group. The true abundance of less prevalent bacteria is always more challenging to detect using (16S rRNA) sequencing. The zero observations in the genus *Intestinibacter* may reflect the true absence of a sub-threshold presence of this genus, which should be confirmed and extended in metagenome sequencing.

We did not replicate the differences in the *Bifidobacterium* genus showing the largest (nominally significant) difference between the ADHD group and controls by Aarts et al., even though this sample overlaps with the current sample (around 40%). There are many methodological reasons contributing to a lack of replication between studies, including DNA extraction [54], 16S rRNA gene region [55], bioinformatic pipeline, data processing and analysis [56], sample size and study design. This is a general problem in the microbiome field, limiting replication of important findings. Follow-up studies (keeping comparable methods and including dietary patterns, comorbid conditions (of ADHD) and bacterial transcriptomics, metabolomics and metagenomics) are needed to replicate the current findings and to understand the complex biological mechanisms underlying our results.

A specifically novel contribution in this dataset is the exploratory comparison between medicated (*N* = 19) and non-medicated individuals (*N* = 22) with ADHD, which showed four genera with a nominally statistically significant lower relative abundance in medicated individuals. The effects of ADHD medication on gut microbiota are very scarce, especially examined at the genus level and in a sample larger than n = four unmedicated ADHD patients as was available in Prehn-Kristensen et al., 2018 [39]. However, the size of these medicated versus unmedicated sub-groups is still small, and hence these results should be interpreted with caution and replicated in larger group samples. Generally, psychotropic medication is found, unintendedly, to have anti-bacterial effects and can alter microbial composition [36]. Research into the effects of ADHD medication on the gut-brain axis in ADHD patients is needed, aiming to dissociate between disease-specific and medication-induced characteristics of the gut microbiota.

This study should be viewed in the context of several strengths and limitations. Our strengths include the use of a sample with high-quality clinical assessment and age-matched clinically ascertained controls. The limitations of our study include (i) limited sample size (although it is the largest sample of its kind so far, *N* = 98) and (ii) lack of information on lifestyle, dietary patterns (including probiotics) or antibiotic use at the time of feces collection. For the former, we applied two QC steps to deal with a large number of variables (genera), their expected small effects and big interindividual variation of the gut microbiota. First, we applied an uncorrected non-parametric approach (to identify the differences between the two groups, reduce the number of variables and prioritize the selection of candidate taxa). Second, we applied an outlier detection step prior to the regression analysis to reduce the chance of false positives/negatives. For the latter, we were only able to collect information on BMI, and while we acknowledge that this is not enough to account for the effects of diet and lifestyle, it is encouraging to see that there was no BMI difference between the groups. Moreover, we looked for and removed samples with a very low bacterial diversity (high proportion of zeros) by applying a 10% genus-based frequency cut-off per sample. This step can be used as a proxy for individuals using antibiotics since they would show a smaller bacterial diversity.

In conclusion, we found subtle, uncorrected differences in the microbiota composition between individuals with ADHD and controls, of which alterations in genera *Prevotella* and *Coprococcus* have also been found by others. Of the three nominally significant different genera, *Coprococcus 2* showed the strongest, though trend level relation with inattention symptoms. Given the scarcity of studies on the gut microbiota in individuals with ADHD, the current results are an important contribution to this field. More studies are needed into the gut microbiota as part of the pathology of ADHD, especially with a bigger sample size across the lifespan and more detailed information about lifestyle. 

## Figures and Tables

**Table 1:** Characteristics of the sample.

	ADHD	Control	Subthreshold ADHD	*p*-Value ^a^
N	41	48	14	-
Age, mean (SD)	20.2 (4.1)	20.4 (3.5)	20.3 (3.4)	NS
Age, range	13–29	13–28	14–26	-
BMI, median (IQR)	23 (20.5–25.5)	22 (20–23)	22 (20–23)	NS
BMI, range	16–31	16–31	20–30	-
BMI ≥ 25, %	29	19	14	NS
Male, %	61	50	36	NS
Use of ADHD medication, N	19	0	3	-
Diff_days, median (IQR)	17 (14–34)	32 (13–64)	14.5 (10.5–30)	NS
Conners’				
Inattention, median (IQR)	64 (58–76)	42 (38–53)	57 (52–64)	<0.001
Hyperactivity/Impulsivity, median (IQR)	56.5 (50–64.5)	41 (36–49)	57 (50–64)	<0.001

^a^ Comparison made for ADHD vs. controls; *t*-test, Mann-Whitney or chi-square test were applied accordingly; one sample had missing value for inattention and hyperactivity/impulsivity scores; four samples had missing value for BMI; four samples were excluded (Figure S1); NS = not significant; SD = standard deviation; IQR = interquartile range; diff_days = represents differences in days between fecal collection and Conner’s assessment.

**Table 2:** Beta diversity analysis.

Variable	N	R^2^	Pseudo-F	*p*-Value
Disorder status	89	0.009	0.79	0.479
Age	103	0.005	0.55	0.727
Sex	103	0.005	0.54	0.750
BMI	98	0.005	0.46	0.874
IA	102	0.009	0.95	0.360
HI	102	0.010	1.04	0.322
medication	41	0.021	0.84	0.469

Results of ADONIS on weighted UniFrac dissimilarity matrix including six tests for disorder status, age, sex, BMI, Inattention (IA) and Hyperactivity/Impulsivity (HI) variables; R^2^ = variance explained, a measure of effect size; Pseudo-F = indicator of the number of clusters, the larger pseudo-F value, the greater between-group variation than the within-group variation.

**Table 3:** Association of the genera with ADHD symptoms scores.

	Inattention				Hyperactivity/Impulsivity			
	N	B (S.E.) ^a^	95% CI	*p*-Value	N	B (S.E.) ^a^	95% CI	*p*-Value
*Prevotella_9*	98	0.111 (0.099)	−0.079–0.306	0.267	98	0.118 (0.096)	−0.065–0.308	0.222
*Coprococcus_2*	95	−3.189 (1.639)	−6.325–(−0.029)	0.055	96	−2.331 (1.456)	−5.108–0.492	0.113
*Intestinibacter*	85	191.161 (139.654)	−74.119–4.587	0.175	94	33.829 (22.779)	−9.855–77.482	0.141

Linear regression models for the relative abundance of the selected genera (based on the Mann-Whitney U test) with the ADHD symptoms scores (inattention and hyperactivity/impulsivity) measured from participants with ADHD and controls and subthreshold ADHD; ^a^ Linear regression model without samples removed based on Cook’s distance and Leverage threshold; models adjusted for age, sex, BMI, diff_days and a random factor for family relatedness. There was no significant association after multiple testing correction (FDR); *N* = number of samples after the removal of outliers (*N* = 98 means no outliers were removed); B = coefficient; S.E. = standard error; CI = Confidence Interval.

## Supplementary Materials

The following are available online at https://www.mdpi.com/2076-2607/8/3/406/s1, include Supplemental Results, Tables and Figures; Figure S1. Filtering procedure applied in our study; Figure S2. Alpha diversity comparison of the gut microbiome between participants with ADHD and controls; Figure S3. Principal coordinates analysis (PCoA) of weighted UniFrac distances representing microbial composition of participants with ADHD and controls; Figure S4. Boxplot of four genera relative abundance being different between medicated and non-medicated participants with ADHD; Figure S5. Spearman’s rank correlation matrix of the three genera being significantly different between participants with ADHD and controls; Table S1. Total number of reads of participants with ADHD, controls and subthreshold ADHD; Table S2. Relative abundance of bacterial phyla of all participants in our study; Table S3. Multiple regression model with the three genera on inattention.

## References

[1] Franke B., Faraone S.V., Asherson P., Buitelaar J., Bau C.H., Ramos-Quiroga J.A., Mick E., Grevet E.H., Johansson S., Haavik J. (2012). The genetics of attention deficit/hyperactivity disorder in adults, a review. Mol. Psychiatry.

[2] Polanczyk G., de Lima M.S., Horta B.L., Biederman J., Rohde L.A. (2007). The worldwide prevalence of ADHD: A systematic review and metaregression analysis. Am. J. Psychiatry.

[3] Fredriksen M., Dahl A.A., Martinsen E.W., Klungsoyr O., Faraone S.V., Peleikis D.E. (2014). Childhood and persistent ADHD symptoms associated with educational failure and long-term occupational disability in adult ADHD. Atten. Defic. Hyperact. Disord..

[4] Adamou M., Arif M., Asherson P., Aw T.C., Bolea B., Coghill D., Guðjónsson G., Halmøy A., Hodgkins P., Müller U. (2013). Occupational issues of adults with ADHD. BMC Psychiatry.

[5] Franke B., Michelini G., Asherson P., Banaschewski T., Bilbow A., Buitelaar J.K., Cormand B., Faraone S.V., Ginsberg Y., Haavik J. (2018). Live fast, die young? A review on the developmental trajectories of ADHD across the lifespan. Eur. Neuropsychopharmacol..

[6] Langley K. (2018). ADHD Genetics. Oxford Textbook of Attention Deficit Hyperactivity Disorder.

[7] Cortese S., Adamo N., Del Giovane C., Mohr-Jensen C., Hayes A.J., Carucci S., Atkinson L.Z., Tessari L., Banaschewski T., Coghill D. (2018). Comparative efficacy and tolerability of medications for attention-deficit hyperactivity disorder in children, adolescents, and adults: A systematic review and network meta-analysis. Lancet Psychiatry.

[8] Childress A.C., Sallee F.R. (2014). Attention-deficit/hyperactivity disorder with inadequate response to stimulants: Approaches to management. CNS Drugs.

[9] O’Callaghan P. (2014). Adherence to stimulants in adult ADHD. Atten. Deficit Hyperact. Disord..

[10] Ghuman J.K., Ghuman H.S. (2013). Pharmacologic intervention for attention-deficit hyperactivity disorder in preschoolers: Is it justified?. Paediatr. Drugs.

[11] De Crescenzo F., Cortese S., Adamo N., Janiri L. (2017). Pharmacological and non-pharmacological treatment of adults with ADHD: A meta-review. Evid. Based Ment. Health.

[12] Ly V., Bottelier M., Hoekstra P.J., Vasquez A.A., Buitelaar J.K., Rommelse N.N. (2017). Elimination diets’ efficacy and mechanisms in attention deficit hyperactivity disorder and autism spectrum disorder. Eur. Child Adolesc. Psychiatry.

[13] Pelsser L.M., Frankena K., Toorman J., Rodrigues Pereira R. (2017). Diet and ADHD, Reviewing the Evidence: A Systematic Review of Meta-Analyses of Double-Blind Placebo-Controlled Trials Evaluating the Efficacy of Diet Interventions on the Behavior of Children with ADHD. PLoS ONE.

[14] Vuong H.E., Yano J.M., Fung T.C., Hsiao E.Y. (2017). The Microbiome and Host Behavior. Annu. Rev. Neurosci..

[15] Hsiao E.Y., McBride S.W., Hsien S., Sharon G., Hyde E.R., McCue T., Codelli J.A., Chow J., Reisman S.E., Petrosino J.F. (2013). Microbiota modulate behavioral and physiological abnormalities associated with neurodevelopmental disorders. Cell.

[16] Bull-Larsen S., Mohajeri M.H. (2019). The Potential Influence of the Bacterial Microbiome on the Development and Progression of ADHD. Nutrients.

[17] Dong W., Wang R., Ma L.N., Xu B.L., Zhang J.S., Zhao Z.W., Wang Y.L., Zhang X. (2016). Influence of age-related learning and memory capacity of mice: Different effects of a high and low caloric diet. Aging Clin. Exp. Res..

[18] Carabotti M., Scirocco A., Maselli M.A., Severi C. (2015). The gut-brain axis: Interactions between enteric microbiota, central and enteric nervous systems. Ann. Gastroenterol..

[19] Aarts E., Ederveen T.H.A., Naaijen J., Zwiers M.P., Boekhorst J., Timmerman H.M., Smeekens S.P., Netea M.G., Buitelaar J.K., Franke B. (2017). Gut microbiome in ADHD and its relation to neural reward anticipation. PLoS ONE.

[20] von Rhein D., Mennes M., van Ewijk H., Groenman A.P., Zwiers M.P., Oosterlaan J., Heslenfeld D., Franke B., Hoekstra P.J., Faraone S.V. (2015). The NeuroIMAGE study: A prospective phenotypic, cognitive, genetic and MRI study in children with attention-deficit/hyperactivity disorder. Design and descriptives. Eur. Child Adolesc. Psychiatry.

[21] Fernandez-Calleja J.M.S., Konstanti P., Swarts H.J.M., Bouwman L.M.S., Garcia-Campayo V., Billecke N., Oosting A., Smidt H., Keijer J., van Schothorst E.M. (2018). Non-invasive continuous real-time in vivo analysis of microbial hydrogen production shows adaptation to fermentable carbohydrates in mice. Sci. Rep..

[22] van den Bogert B., Erkus O., Boekhorst J., de Goffau M., Smid E.J., Zoetendal E.G., Kleerebezem M. (2013). Diversity of human small intestinal Streptococcus and Veillonella populations. FEMS Microbiol. Ecol..

[23] Ramiro-Garcia J., Hermes G.D.A., Giatsis C., Sipkema D., Zoetendal E.G., Schaap P.J., Smidt H. (2016). NG-Tax, a highly accurate and validated pipeline for analysis of 16S rRNA amplicons from complex biomes. F1000Research.

[24] Quast C., Pruesse E., Yilmaz P., Gerken J., Schweer T., Yarza P., Peplies J., Glockner F.O. (2013). The SILVA ribosomal RNA gene database project: Improved data processing and web-based tools. Nucleic Acids Res..

[25] Morris E.K., Caruso T., Buscot F., Fischer M., Hancock C., Maier T.S., Meiners T., Muller C., Obermaier E., Prati D. (2014). Choosing and using diversity indices: Insights for ecological applications from the German Biodiversity Exploratories. Ecol. Evol..

[26] Faith D.P. (1992). Conservation evaluation and phylogenetic diversity. Biol. Conserv..

[27] Ernst F.G.M., Huang R., Shetty S., Borman T., Braccia D.C., Bravo H.C., Lahti L. Microbiome R Package. http://microbiome.github.io.

[28] Ursell L.K., Metcalf J.L., Parfrey L.W., Knight R. (2012). Defining the human microbiome. Nutr. Rev..

[29] Anderson M.J. (2001). A new method for non-parametric multivariate analysis of variance. Austral Ecol..

[30] Anderson M.J., Ellingsen K.E., McArdle B.H. (2006). Multivariate dispersion as a measure of beta diversity. Ecol. Lett..

[31] Anderson M.J. (2006). Distance-based tests for homogeneity of multivariate dispersions. Biometrics.

[32] Paul J. (2013). McMurdie and Susan Holmes. phyloseq: An R package for reproducible interactive analysis and graphics of microbiome census data. PLoS ONE.

[33] Kitchen C.M. (2009). Nonparametric vs parametric tests of location in biomedical research. Am. J. Ophthalmol..

[34] Altman N., Krzywinski M. (2016). Analyzing outliers: Influential or nuisance?. Nat. Methods.

[35] Field A. (2009). Discovering Statistics Using SPSS: (And Sex and Drugs and Rock ‘n’ Roll).

[36] Cussotto S., Strain C.R., Fouhy F., Strain R.G., Peterson V.L., Clarke G., Stanton C., Dinan T.G., Cryan J.F. (2019). Differential effects of psychotropic drugs on microbiome composition and gastrointestinal function. Psychopharmacology.

[37] Maier L., Pruteanu M., Kuhn M., Zeller G., Telzerow A., Anderson E.E., Brochado A.R., Fernandez K.C., Dose H., Mori H. (2018). Extensive impact of non-antibiotic drugs on human gut bacteria. Nature.

[38] D’Argenio V., Salvatore F. (2015). The role of the gut microbiome in the healthy adult status. Clin. Chim. Acta.

[39] Prehn-Kristensen A., Zimmermann A., Tittmann L., Lieb W., Schreiber S., Baving L., Fischer A. (2018). Reduced microbiome alpha diversity in young patients with ADHD. PLoS ONE.

[40] Jiang H.-Y., Zhou Y.-Y., Zhou G.-L., Li Y.-C., Yuan J., Li X.-H., Ruan B. (2018). Gut microbiota profiles in treatment-naïve children with attention deficit hyperactivity disorder. Behav. Brain Res..

[41] Akram H. (2017). Characterizing a Link between Gut Microbiome and Attention Deficit Hyperactive Disorder. Honors College Research Collection.

[42] Wang L.-J., Yang C.-Y., Chou W.-J., Lee M.-J., Chou M.-C., Kuo H.-C., Yeh Y.-M., Lee S.-Y., Huang L.-H., Li S.-C. (2019). Gut microbiota and dietary patterns in children with attention-deficit/hyperactivity disorder. Eur. Child Adolesc. Psychiatry.

[43] Tengeler A.C., Dam S.A., Wiesmann M., Naaijen J., Van Bodegom M., Belzer C., Dederen P.J., Verweij V., Franke B., Kozicz T. (2020). Gut microbiota from persons with attention-deficit/hyperactivity disorder affects the brain in mice. Microbiome.

[44] Koh A., De Vadder F., Kovatcheva-Datchary P., Bäckhed F. (2016). From Dietary Fiber to Host Physiology: Short-Chain Fatty Acids as Key Bacterial Metabolites. Cell.

[45] den Besten G., van Eunen K., Groen A.K., Venema K., Reijngoud D.J., Bakker B.M. (2013). The role of short-chain fatty acids in the interplay between diet, gut microbiota, and host energy metabolism. J. Lipid Res..

[46] Dam S.A., Mostert J.C., Szopinska-Tokov J.W., Bloemendaal M., Amato M., Arias-Vasquez A. (2019). The Role of the Gut-Brain Axis in Attention-Deficit/Hyperactivity Disorder. Gastroenterol. Clin. N. Am..

[47] Wang L., Christophersen C.T., Sorich M.J., Gerber J.P., Angley M.T., Conlon M.A. (2012). Elevated Fecal Short Chain Fatty Acid and Ammonia Concentrations in Children with Autism Spectrum Disorder. Dig. Dis. Sci..

[48] Abdelli L.S., Samsam A., Naser S.A. (2019). Propionic Acid Induces Gliosis and Neuro-inflammation through Modulation of PTEN/AKT Pathway in Autism Spectrum Disorder. Sci. Rep..

[49] Gerritsen J., Fuentes S., Grievink W., Van Niftrik L., Tindall B.J., Timmerman H.M., Rijkers G.T., Smidt H. (2014). Characterization of Romboutsia ilealis gen. nov., sp. nov., isolated from the gastro-intestinal tract of a rat, and proposal for the reclassification of five closely related members of the genus Clostridium into the genera Romboutsia gen. nov., Intestinibacter gen. nov., Terrisporobacter gen. nov. and Asaccharospora gen. nov. Int. J. Syst. Evol. Microbiol..

[50] Forslund K., Hildebrand F., Nielsen T.R., Falony G., Le Chatelier E., Sunagawa S., Prifti E., Vieira-Silva S., Gudmundsdottir V., Pedersen H.K. (2015). Disentangling type 2 diabetes and metformin treatment signatures in the human gut microbiota. Nature.

[51] Paone P., Cani P.D. (2020). Mucus barrier, mucins and gut microbiota: The expected slimy partners?. Gut.

[52] Wasilewska J.J., Klukowski M. (2015). Gastrointestinal symptoms and autism spectrum disorder: Links and risks—A possible new overlap syndrome. Pediatr. Health Med. Ther..

[53] Chen M.H., Su T.P., Chen Y.S., Hsu J.W., Huang K.L., Chang W.H., Bai Y.M. (2017). Comorbidity of Allergic and Autoimmune Diseases among Patients with ADHD: A Nationwide Population-Based Study. J. Atten. Disord..

[54] Szopinska J.W., Gresse R., Van Der Marel S., Boekhorst J., Lukovac S., Van Swam I., Franke B., Timmerman H., Belzer C., Vasquez A.A. (2018). Reliability of a participant-friendly fecal collection method for microbiome analyses: A step towards large sample size investigation. BMC Microbiol..

[55] Rintala A., Pietilä S., Munukka E., Eerola E., Pursiheimo J.-P., Laiho A., Pekkala S., Huovinen P. (2017). Gut Microbiota Analysis Results Are Highly Dependent on the 16S rRNA Gene Target Region, Whereas the Impact of DNA Extraction Is Minor. J. Biomol. Tech..

[56] Allali I., Arnold J.W., Roach J., Cadenas M.B., Butz N., Hassan H.M., Koci M., Ballou A., Mendoza M., Ali R. (2017). A comparison of sequencing platforms and bioinformatics pipelines for compositional analysis of the gut microbiome. BMC Microbiol..

