# Correction: Szopinska-Tokov et al. Investigating the Gut Microbiota Composition of Individuals with Attention-Deficit/Hyperactivity Disorder and Association with Symptoms. *Microorganisms* 2020, *8*, 406

**DOI:** 10.3390/microorganisms9071358

**Published:** 2021-06-23

**Authors:** Joanna Szopinska-Tokov, Sarita Dam, Jilly Naaijen, Prokopis Konstanti, Nanda Rommelse, Clara Belzer, Jan Buitelaar, Barbara Franke, Mirjam Bloemendaal, Esther Aarts, Alejandro Arias Vasquez

**Affiliations:** 1 Department of Psychiatry, Radboudumc, Donders Institute for Brain, Cognition and Behaviour, 6525 GA Nijmegen, The Netherlands; Joanna.Szopinska-Tokov@radboudumc.nl (J.S.-T.); Nanda.Lambregts-Rommelse@radboudumc.nl (N.R.); Barbara.Franke@radboudumc.nl (B.F.); Mirjam.Bloemendaal@radboudumc.nl (M.B.); 2 Department of Cognitive Neuroscience, Radboudumc, Donders Institute for Brain, Cognition and Behaviour, 6525 EN Nijmegen, The Netherlands; Sarita.Dam@radboudumc.nl (S.D.); jilly.naaijen@donders.ru.nl (J.N.); Jan.Buitelaar@radboudumc.nl (J.B.); 3 Laboratory of Microbiology, Wageningen University, 6708 WE Wageningen, The Netherlands; prokopis.konstanti@wur.nl (P.K.); clara.belzer@wur.nl (C.B.); 4 Karakter Child and Adolescent Psychiatry University Center, 6525 GC Nijmegen, The Netherlands; 5 Department of Human Genetics, Radboudumc, Donders Institute for Brain, Cognition and Behaviour, 6525 GA Nijmegen, The Netherlands; 6 Centre for Cognitive Neuroimaging, Donders Institute for Brain, Cognition and Behaviour, Radboud University, 6525 EN Nijmegen, The Netherlands; esther.aarts@donders.ru.nl

The authors wish to make the following correction to this paper [1]:

After the publication of the manuscript, the authors recognized a mismatch in the link between the microbiota sequencing data (from ADHD cases and controls) and their descriptive and behavioral data. Thus, the manuscript had to be reanalyzed and rewritten, resulting in different results and conclusion. The main difference is that the case-control comparison resulted in different bacteria differences. Moreover, we did not find an association (only at trend level) between the microbiome relative abundance and inattention score. The corrected results, discussion, and conclusion, can be found below. Due to the changes the abstract, and material and methods section had to be adjusted as well. The changes are provided below. 

The authors would like to apologize for any inconvenience caused to the readers by these changes.

## Changes in Abstract

The results and conclusion in the abstract changed to:

Alpha and Beta-diversity were not different between participants with ADHD and healthy controls. Three genera showed nominal differences (*p*_uncorrected_ < 0.05) between both groups (*Prevotella_9*, *Coprococcus_2* and *Intestinibacter*) and were further tested for their association with ADHD symptom scores (adjusting for age, sex, body mass index, a time delay between feces collection and symptoms assessment, medication use and family relatedness). Our results show that the variation of a genus from the *Lachnospiraceae* family (*Coprococcus_2*) showed a trend of being negatively associated with inattention symptoms. Furthermore, we showed that the relative abundance of four genera was reduced by ADHD medication (*p*_uncorrected_ < 0.05). Overall, our results may support the role of the gut microbiota in the pathophysiology of ADHD. Given the scarcity of studies on the gut microbiota in individuals with ADHD, the current results are an important contribution to this field. More studies are needed into the gut microbiota as part of the pathology of ADHD, especially with a bigger sample size across the lifespan and more detailed information about lifestyle.

## Changes in Materials and Methods

Certain changes had to be applied in the material and methods section. First, for easier maintenance and reproducibility, we used R software instead of SPSS to reanalyze microbiome data. This means that we calculated the alpha-diversity metrics using the R function microbiome::alpha (version 1.6.0) and the composition analysis using “phyloseq” R package version 1.28.0. Second, we used the nonparametric Mann-Whitney U test method in order to identify differences in genera between cases and controls. This was visualized by using a boxplot with a summary table representing the number of zeros using “ggpubr” R package version 0.4.0.999. Third, in the regression analyses, we had to adjust the number of total tests used in FDR to 6 and not 14 tests. Fourth, the new results of the “2.2.9. Correlation Analysis and Multiple Regression with All Selected Genera” are shown in the Supplementary Materials. 

## Changes in Results

The data had to be reanalyzed; thus, all the results changed includes all the tables and figures. For easier readability, the whole (corrected) results section is provided below: 

## 3. Results

### 3.1. Subjects Characteristics

The general characteristics of the studied sample are presented in Table 1. Mean age, median BMI, percentage of males, and differences in days between fecal collection and ADHD symptoms assessment (diff_days) were similar among the two groups. As expected, mean inattention and hyperactivity/impulsivity scores were statistically different between the ADHD and control groups. Out of the 41 participants with ADHD, 19 were using medication for ADHD. 

### 3.2. Microbiota Measures

*Within- and between-sample diversity metrics*: None of the three alpha-diversity (within-sample diversity) measures showed significant differences between the ADHD and control groups (Figure S2).

Beta-diversity (between-sample diversity), assessed using betadisper [2], showed that the ADHD group had a smaller variation in the gut microbiota composition (*p* = 0.08; Figure 1 and Figure S3). PCoA based on weighted UniFrac distance did not show discrimination of microbial composition between the two groups determined by disorder status (ADHD vs. controls) (Figure S3). This was supported by the statistical test—ADONIS, where participants with ADHD and controls samples displayed non-significant separation according to weighted UniFrac distance (variance explained = 0.9%, *p* = 0.479, N = 89). Other variables, such as age, sex, BMI, inattention score (IA), hyperactivity-impulsivity score (HI), and medication, did not show a significant effect on beta-diversity (Table 2). 

#### 3.2.1. Taxonomic Composition Analysis and Associations with Symptoms

As expected from [3], a compositional analysis of our samples revealed that *Firmicutes*, *Bacteroidetes*, *Actinobacteria*, *Proteobacteria*, and *Verrucomicrobia*, were the most frequent phyla in our data (Table S2). There were no significant differences in the relative abundance of any of these phyla between participants with ADHD and controls (Table S2).

At the genus level, differences in the gut microbiota composition revealed nominal significant case-control differences for three genera (*p* < 0.05; Figure 2). Of those, one genus was higher, and two were lower in participants with ADHD compared with control samples. One genus, *Coprococcus_2* showed a trend of being negatively associated (B = (−3.189), *p* = 0.055, Q = 0.33; corrected for multiple testing; Table 3) with inattention scores. We did not find any association between tested genera and hyperactivity/impulsivity scores (before or after correcting for multiple testing; all *p* > 0.05); therefore, only IA was considered in further analyses.

#### 3.2.2. Effect of Medication on the Regression Results and on Gut Microbiota Composition

We tested the effect of ADHD medication on the (regression) results by excluding medicated cases (N = 19) from the analysis. We found that medication reduced the beta coefficient from −3.189 to −2.806 in the association between *Coprococcus_2* and symptoms of inattention (B = (−2.806), *p* = 0.080 vs. results in Table 3). This reduction can be due to the reduction in sample size (N = 79 vs. N = 95).

We performed a post hoc exploratory analysis where we compared the relative abundance of all the genera (total taxa compared = 77) between the medicated (N = 19) vs. non-medicated (N = 22) individuals with ADHD. We found that four genera (*Lactobacillus*, *Lachnospiraceae_ND3007_group*, *Ruminococcaceae_g__* and *Ruminococcaceae_UCG.014*) were decreased in medicated ADHD (*p*_uncorrected_≤ 0.05; Figure S4). Regarding the *Lactobacillus* results, we had to treat them with caution because we only had three non-zero values for medicated cases.

## Changes in Discussion

Due to reanalyzed data and a change in results, the discussion and conclusion were adjusted accordingly. For easier readability, the whole (corrected) discussion section is provided below:

## 4. Discussion

This study aimed to determine the differences in gut microbiota composition between individuals with ADHD and controls and the association between the abundance of the selected genera and the severity of ADHD symptoms (inattention and hyperactivity/impulsivity) accounting for the effects of medication. Our results did not show general differences in microbiota composition (beta-diversity) between the groups. At the taxonomic level, we found nominal (uncorrected significant) differences at the genus level; lower abundance of *Prevotella_9* and *Coprococcus_2* and higher abundance of *Intestinibacter* in individuals with ADHD compared to controls. Of these three genera, *Coproccocus_2* related most strongly (*p* = 0.055) with ADHD symptoms, specifically Inattention symptoms. Excluding subjects that were using ADHD medication from the regression model slightly reduced the strength of the association. Together this indicates that differences in gut microbiome in this sample of ADHD patients compared with control subjects are subtle.

Our results align with the growing evidence that gut microbiome alterations might be part of the pathology of ADHD [4,5,6,7,8]. The taxa, observed to be nominally different, partly overlap with previous findings. For example, while not the genus showing the largest differences, Aarts et al. also found the genus *Coprococcus* to be underrepresented in individuals with ADHD [6]. Our lab recently performed a humanization study, in which six randomly selected microbiome samples from the NeuroIMAGE cohort (the cohort studied here) were transplanted into germ-free wild-type mice [9]. Mice colonized with ADHD gut microbiota had increased anxiety-like behavior and showed significantly altered structural and functional brain characteristics. When comparing taxonomy between cases and controls in this humanization approach, again, *Coprococcus_2* was found altered. Here, the effect was in the opposing direction; relative abundance was increased in mice colonized with ADHD gut microbiota, wherein the current case-control comparison *Coprococcus_2* abundance was higher in controls. Putting aside differences in the directions of effects, the fact that genus *Coporococcus_2* surfaces in both case-control comparisons suggest that this is an interesting target for replication in gut microbiota associated with ADHD diagnosis.

Furthermore, an abundance of the genus *Prevotella* was also found lower in children with ADHD compared with controls [4]. Functionally, *Prevotella* spp. and some *Coprococcus* species have been identified as short-chain fatty acids (SCFAs) producers [10], which can be absorbed and used as an energy source by the host [11]. SCFAs producers have been shown to play a potential role in ADHD [12] and autism [13,14] through several of the gut-brain-routes, including their anti-inflammatory effects on the central nervous system.

The only genus with a higher rather than lower relative abundance in cases versus controls, *Intestinibacter* (belonging to *Peptostreptococcaceae*), was defined only recently [15]; not much is known about its role in ADHD and human health in general. A potential function may be involved in mucus degradation [16]. Mucus-degrading bacteria are linked to inflammatory bowel disease [17], a comorbid diagnosis seen in neurodevelopmental disorders like ASD [18] or ADHD [19]. Note that the relative abundance of this genus is quite low in both groups, and the statistical difference is based on ten non-zero observations in the ADHD group versus two non-zero observations in the control group. The true abundance of less prevalent bacteria is always more challenging to detect using (16S rRNA) sequencing. The zero observations in the genus *Intestinibacter* may reflect the true absence of a sub-threshold presence of this genus, which should be confirmed and extended in metagenome sequencing.

We did not replicate the differences in the *Bifidobacterium* genus showing the largest (nominally significant) difference between the ADHD group and controls by Aarts et al., even though this sample overlaps with the current sample (around 40%). There are many methodological reasons contributing to a lack of replication between studies, including DNA extraction [20], 16S rRNA gene region [21], bioinformatic pipeline, data processing and analysis [22], sample size and study design. This is a general problem in the microbiome field, limiting replication of important findings. Follow-up studies (keeping comparable methods and including dietary patterns, comorbid conditions (of ADHD) and bacterial transcriptomics, metabolomics and metagenomics) are needed to replicate the current findings and to understand the complex biological mechanisms underlying our results.

A specifically novel contribution in this dataset is the exploratory comparison between medicated (N = 19) and non-medicated individuals (N = 22) with ADHD, which showed four genera with a nominally statistically significant lower relative abundance in medicated individuals. The effects of ADHD medication on gut microbiota are very scarce, especially examined at the genus level and in a sample larger than n = four unmedicated ADHD patients as was available in Prehn-Kristensen et al., 2018 [4]. However, the size of these medicated versus unmedicated sub-groups is still small, and hence these results should be interpreted with caution and replicated in larger group samples. Generally, psychotropic medication is found, unintendedly, to have anti-bacterial effects and can alter microbial composition [23]. Research into the effects of ADHD medication on the gut-brain axis in ADHD patients is needed, aiming to dissociate between disease-specific and medication-induced characteristics of the gut microbiota.

This study should be viewed in the context of several strengths and limitations. Our strengths include the use of a sample with high-quality clinical assessment and age-matched clinically ascertained controls. The limitations of our study include (i) limited sample size (although it is the largest sample of its kind so far, N = 98) and (ii) lack of information on lifestyle, dietary patterns (including probiotics) or antibiotic use at the time of feces collection. For the former, we applied two QC steps to deal with a large number of variables (genera), their expected small effects and big interindividual variation of the gut microbiota. First, we applied an uncorrected non-parametric approach (to identify the differences between the two groups, reduce the number of variables and prioritize the selection of candidate taxa). Second, we applied an outlier detection step prior to the regression analysis to reduce the chance of false positives/negatives. For the latter, we were only able to collect information on BMI, and while we acknowledge that this is not enough to account for the effects of diet and lifestyle, it is encouraging to see that there was no BMI difference between the groups. Moreover, we looked for and removed samples with a very low bacterial diversity (high proportion of zeros) by applying a 10% genus-based frequency cut-off per sample. This step can be used as a proxy for individuals using antibiotics since they would show a smaller bacterial diversity.

In conclusion, we found subtle, uncorrected differences in the microbiota composition between individuals with ADHD and controls, of which alterations in genera *Prevotella* and *Coprococcus* have also been found by others. Of the three nominally significant different genera, *Coprococcus 2* showed the strongest, though trend level relation with inattention symptoms. Given the scarcity of studies on the gut microbiota in individuals with ADHD, the current results are an important contribution to this field. More studies are needed into the gut microbiota as part of the pathology of ADHD, especially with a bigger sample size across the lifespan and more detailed information about lifestyle.

## Change in Supplementary Materials

The Supplementary Materials were changed accordingly and were included as a separate document.

## Change in Author Names (Add a New One)

Due to applied changes, we would like to add Mirjam Bloemendaal as a co-author in order to emphasize her significant contribution to this correction; this was approved by all co-authors. She should be recognized for her help in verifying the applied changes, as well as in structuring and writing the correction paper.

## Figures and Tables

**Figure 1:**
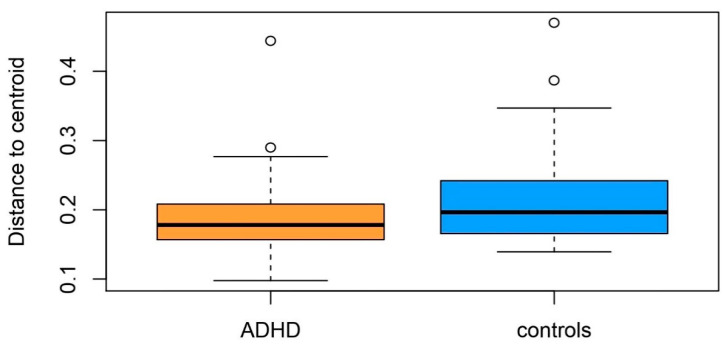
Boxplot of multivariate homogeneity of groups’ dispersions (betadisper) of participants with ADHD and controls. Box plots represent median with whiskers on ±1.5 IQR. Pseudo-F = 3.051, *p* = 0.08.

**Figure 2:**
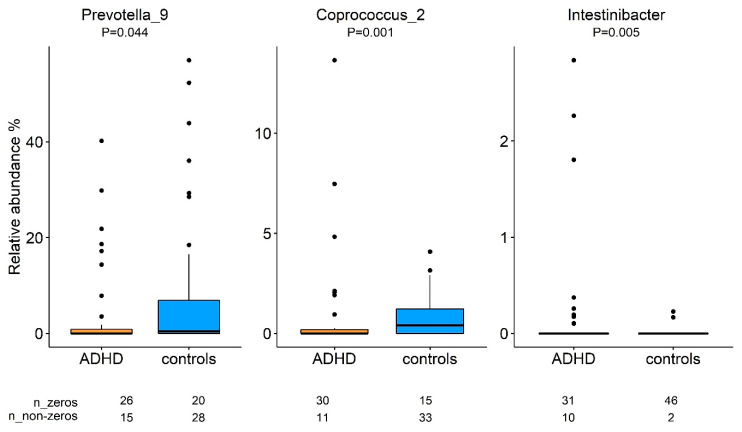
Comparison of bacterial relative abundance between participants with ADHD and controls. Identification of the bacteria differences was made by the Mann-Whitney test. No outliers were removed since we used a non-parametric test which is less sensitive to the extreme values. Box plots represent median with whiskers on ±1.5 IQR. Nominal significant threshold: *p* < 0.05.

**Table 1:** Characteristics of the sample.

	ADHD	Control	Subthreshold ADHD	*p*-Value ^a^
N	41	48	14	-
Age, mean (SD)	20.2 (4.1)	20.4 (3.5)	20.3 (3.4)	NS
Age, range	13–29	13–28	14–26	-
BMI, median (IQR)	23 (20.5–25.5)	22 (20–23)	22 (20–23)	NS
BMI, range	16–31	16–31	20–30	-
BMI ≥ 25, %	29	19	14	NS
Male, %	61	50	36	NS
Use of ADHD medication, N	19	0	3	-
Diff_days, median (IQR)	17 (14–34)	32 (13–64)	14.5 (10.5–30)	NS
**Conners’**				
Inattention, median (IQR)	64 (58–76)	42 (38–53)	57 (52–64)	<0.001
Hyperactivity/Impulsivity, median (IQR)	56.5 (50–64.5)	41 (36–49)	57 (50–64)	<0.001

^a^ Comparison made for ADHD vs. controls; *t*-test, Mann-Whitney or chi-square test were applied accordingly; one sample had missing value for inattention and hyperactivity/impulsivity scores; four samples had missing value for BMI; four samples were excluded (Figure S1); NS = not significant; SD = standard deviation; IQR = interquartile range; diff_days = represents differences in days between fecal collection and Conner’s assessment.

**Table 2:** Beta diversity analysis.

Variable	N	R^2^	Pseudo-F	*p*-Value
Disorder status	89	0.009	0.79	0.479
Age	103	0.005	0.55	0.727
Sex	103	0.005	0.54	0.750
BMI	98	0.005	0.46	0.874
IA	102	0.009	0.95	0.360
HI	102	0.010	1.04	0.322
medication	41	0.021	0.84	0.469

Results of ADONIS on weighted UniFrac dissimilarity matrix including six tests for disorder status, age, sex, BMI, Inattention (IA) and Hyperactivity/Impulsivity (HI) variables; R^2^ = variance explained, a measure of effect size; Pseudo-F = indicator of the number of clusters, the larger pseudo-F value, the greater between-group variation than the within-group variation.

**Table 3:** Association of the genera with ADHD symptoms scores.

	Inattention				Hyperactivity/Impulsivity			
	N	B (S.E.) ^a^	95% CI	*p*-Value	N	B (S.E.) ^a^	95% CI	*p*-Value
*Prevotella_9*	98	0.111 (0.099)	−0.079–0.306	0.267	98	0.118 (0.096)	−0.065–0.308	0.222
*Coprococcus_2*	95	−3.189 (1.639)	−6.325–(−0.029)	0.055	96	−2.331 (1.456)	−5.108–0.492	0.113
*Intestinibacter*	85	191.161 (139.654)	−74.119–4.587	0.175	94	33.829 (22.779)	−9.855–77.482	0.141

Linear regression models for the relative abundance of the selected genera (based on the Mann-Whitney U test) with the ADHD symptoms scores (inattention and hyperactivity/impulsivity) measured from participants with ADHD and controls and subthreshold ADHD; ^a^ Linear regression model without samples removed based on Cook’s distance and Leverage threshold; models adjusted for age, sex, BMI, diff_days and a random factor for family relatedness. There was no significant association after multiple testing correction (FDR); N = number of samples after the removal of outliers (N = 98 means no outliers were removed); B = coefficient; S.E. = standard error; CI = Confidence Interval.
