# Supplementary material for: Investigating the Gut Microbiota Composition of Individuals with Attention-Deficit/Hyperactivity Disorder and Association with Symptoms
Source: Microorganisms. 2020 Mar 13;8(3):406. doi: 10.3390/microorganisms8030406 (PMC7143990; doi:10.3390/microorganisms8030406)
Supplement: Supplementary file 1 [file microorganisms-08-00406-s001.zip › microorganisms-1223051-supplementary.pdf]

Supplemental Results:

Results of 16S rRNA analysis after the filtering procedure of taxonomic data

One sample was removed after NG-Tax pipeline because of its low number of final total reads. The NG-Tax sequencing results were represented by a total of 1 700 925 reads corresponding to 2 186 OTUs representing 176 genera (Figure S1). After filtering, the taxonomic data was represented by 1 659 853 reads related to 1 965 OTUs within 77 genera. Removed genera (56.25%) were represented by 2.4% of the total reads and 0.18% of total relative abundance. One sample was removed due to having less than 10% of the total observed genera.

Sequencing depth comparison

After filtering of the taxonomic data, the total reads were compared between the groups. This comparison showed that the sequencing depth was evenly distributed among all groups; ADHD (mean $rank_{ADHD}$=46.54; $Median_{ADHD}$=12 489), controls (mean $rank_{controls}$=57.12; $Median_{controls}$=15 477) and subthreshold ADHD (mean $rank_{subtreshold}$=50.43; $Median_{subtreshold}$=14 792; H=2.82, P=0.244) (Table S1).

Correlation analysis and multiple regression with all selected genera

Spearman correlation analysis showed that *Intestinibacter* and *Prevotella_9,* and *Intestinibacter* and *Coprococcus_2* had a weak negative correlation (0.30>r>0.10). *Coprococcus_2* and *Prevotella_9* showed no significant correlation (Figure S5). We carried out a multiple regression analysis including all selected genera in one model (Table S3) and investigated the unique contribution of the associated genera to inattention symptoms. After controlling for other bacterial taxa (and age, sex, BMI, diff_days and a random factor for family

relatedness), the beta coefficient was reduced from -3.189 (P=0.055) to -2.338 (P=0.147) in association between *Coprococcus_2* and symptoms of inattention.

Supplemental Figures and Tables

Figure S1. Filtering procedure applied in our study.

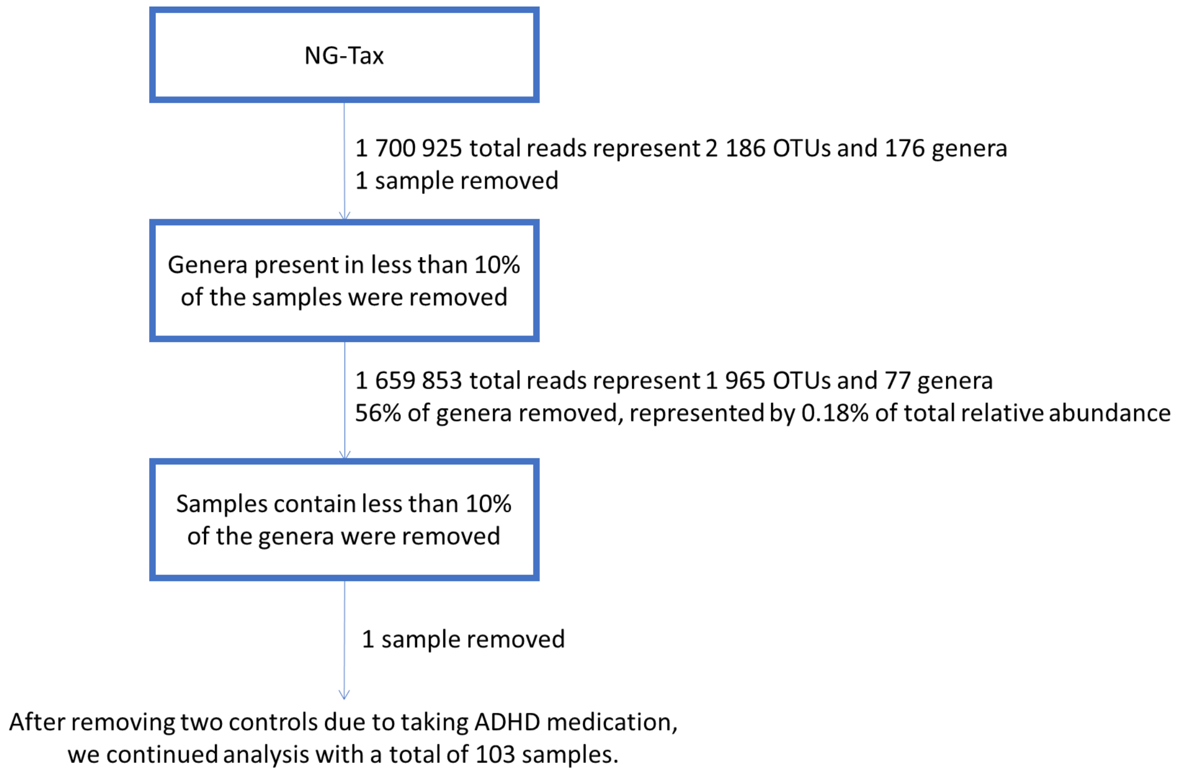

Figure S2. Alpha diversity comparison of the gut microbiome between participants with ADHD and controls. Alpha diversity was indicated by observed OTUs (P=0.087), Shannon index (P=0.81), and Phylogenetic diversity (P=0.59). Box plots represent the median with whiskers on ±1.5 IQR.

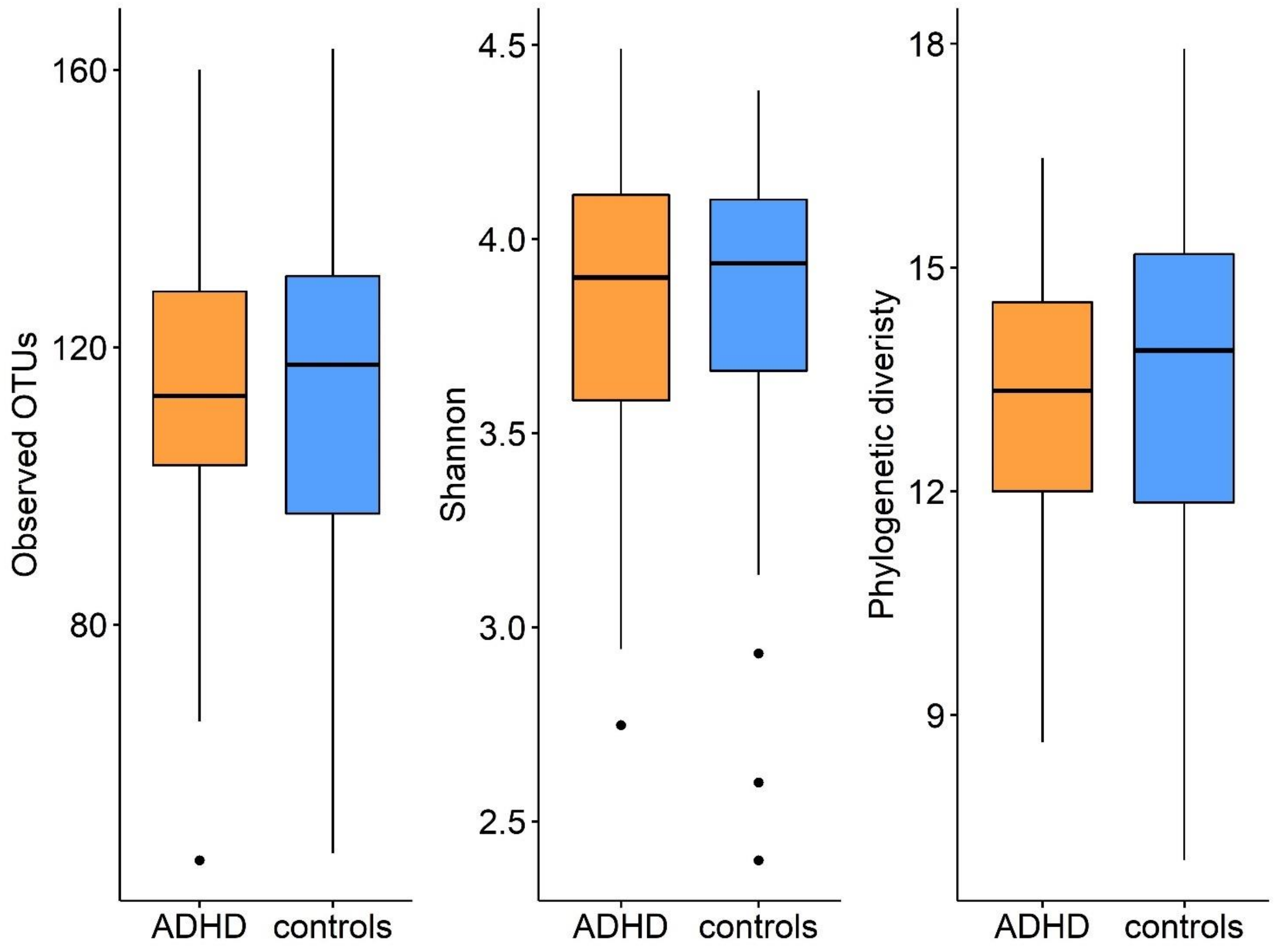

Figure S3. Principal coordinates analysis (PCoA) of weighted UniFrac distances representing the microbial composition of participants with ADHD and controls. The first two components are plotted with the percentage of the variance explained by each principal component. Each point represents an individual sample.

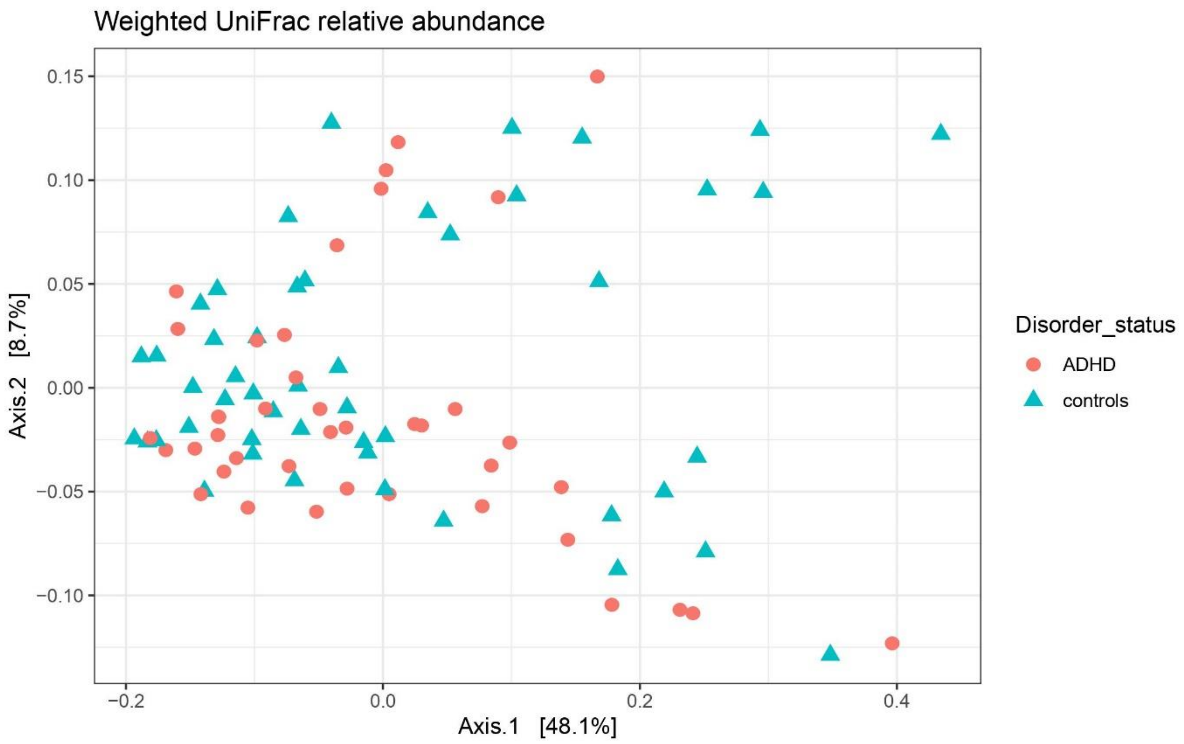

Figure S4. Boxplot of four genera relative abundance being different between medicated and non-medicated participants with ADHD. Box plots represent median with whiskers on ±1.5 IQR.

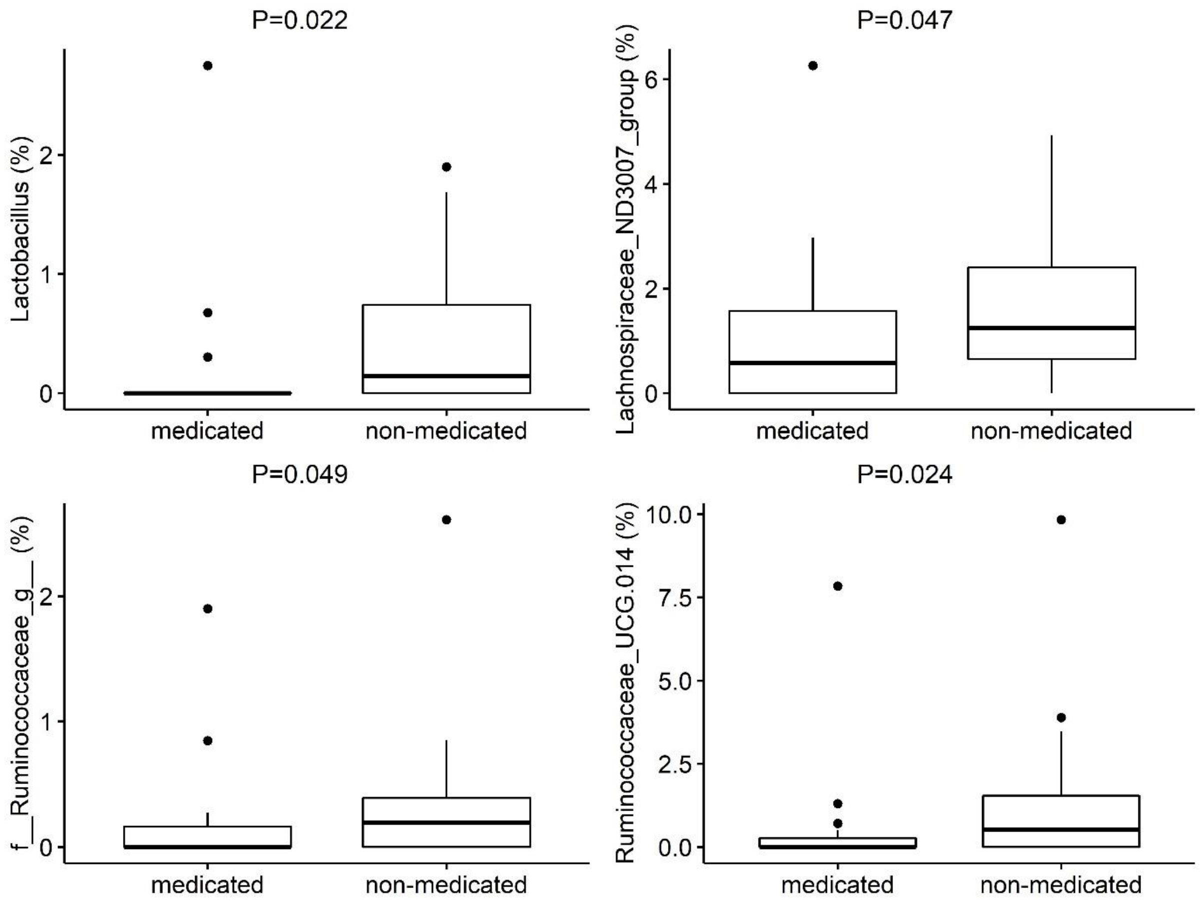

Figure S5. Spearman's rank correlation matrix of the three genera being significantly different between participants with ADHD and controls. The matrix represents only nominal significant (P<0.05) correlations.

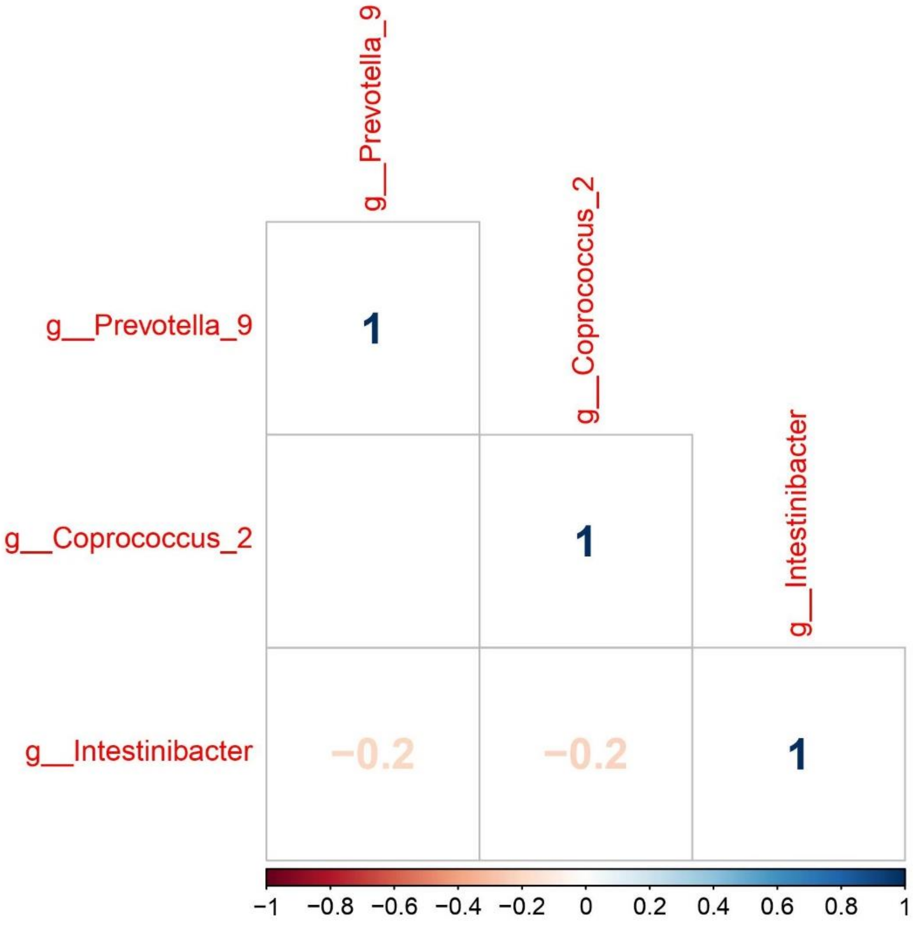

Table S1. Total number of reads of participants with ADHD, controls, and subthreshold ADHD.

	Total number of reads			
	N	Median (IQR)	Mean rank[a]	P-value[a]
ADHD	41	12489 (5712 – 19771)	46.54	
controls	48	15477 (9246 – 21510)	57.12	.244
subthreshold ADHD	14	14792 (3171 - 29660)	50.43	

[a] calculated based on the Kruskal-Wallis Test

Table S2. Relative abundance of bacterial phyla of all participants in our study.

	Phylum's relative abundance and FBR				
	ADHD median (IQR)	ADHD Mean rank[a]	controls median (IQR)	controls Mean rank[a]	P-values[a]
Firmicutes	78.40 (66.0 – 85.40)	45.46	79.60 (61.40 – 85.20)	44.60	0.880
Bacteroidetes	18.20 (9.67 – 32.60)	45.20	16.60 (8.98– 37.40)	44.83	0.951
Actinobacteria	0.84 (0.20 – 2.41)	47.93	0.51 (0.12 – 1.48)	42.50	0.324
Proteobacteria	0.15 (0 – 0.48)	42.82	0.22 (0 – 0.63)	46.86	0.453
Verrucomicrobia	0 (0 – 0.34)	43.00	0.13 (0 – 0.45)	46.71	0.476
FBR	4.44 (2.01 – 8.83)	44.76	4.75 (1.65 – 10.0)	45.21	0.938

N=89; [a] calculated based on the Mann-Whitney U test; FBR = *Firmicutes* to *Bacteroidetes* ratio; IQR = interquartile range.

Table S3. Multiple regression model with the three genera on inattention.

	Inattention			Results taken from Table3[b]	
	B (S.E.)	95% CI	P-value	B (S.E.)	P-value
Prevotella_9	0.112 (0.094)	-0.069 - 0.305	0.240	0.111 (0.099)	0.267
Coprococcus_2	-2.338 (1.595)	-5.327 - 0.772	0.147	-3.189 (1.639)	0.055
Intestinibacter	183.418 (138.238)	-75.846 - 445.689	0.189	191.161 (139.654)	0.175

The model includes all the three genera in one model; The identified outliers were removed from the analysis, N=87; The model was adjusted for age, sex, BMI, diff_days, and a random factor for family relatedness; [b] Results from simple regression models (taken from Table 3 for easier comparison).
